# Abstract of a Noteworthy Presidential Address

**Published:** 1920-08-07

**Authors:** 


### FEDERATION OF MEDICAL & ALLIED SOCIETIES.

### Abstract of a Noteworthy Presidential Address.

Below we print an abstract of the excellent Presidential address delivered by Sir Malcolm Morris at the recent annual meeting of the above Federation. The complete annual report and full details of all the speeches made at the time of its presentation are published in the contemporary medical journals, but in the abstract which follows we give deserved prominence to certain sections which have a particularly important bearing upon the medical politics of the future.

We are not far from the beginning of the great task to which we have put our hands, but the rapid accession to our ranks of numbers of the medical and kindred organisations which it is our aim to bring together for counsel and common action warrants the belief that our movement is destined to ultimate triumph. Our work has not been favoured by the heavy costs of all the facilities of communication. But this difficulty has been reduced to a minimum by the rigid economy that has been practised. So careful have your officers been that the expenditure for the first year has been kept within the estimate by more than £300, and their ingenuity has enabled them so to lay their plans that during the second year, without loss of efficiency, the expenditure will be still further reduced. That so much progress should have been achieved at so small an outlay is a most gratifying circumstance.

### The Evolution of the Federation.

That the evolution of this Federation conforms with the tendencies of the times is sufficiently obvious. The social instinct which prompts corporations to band themselves together for the attainment of common ends has been greatly stimulated as a result of the war. This, indeed, more than anything else, is the keynote of the national reconstruction which is now in progress. Alike in commerce and in industry we see a closing up of the ranks. Business corporations are coalescing. Groups of employers on the one hand and the great trade unions on the other hand are forming alliances that they may enjoy the mutual advantages of united action. Even the nations themselves are striving to form themselves into a league as the one way of saving civilisation from shipwreck. Is it surprising, then, that the medical profession should strive after a federation of the many organisations which represent its sectional interests, and should hold out the hand of fellowship to the bodies which represent the kindred professions?

The need for such a Federation can be deduced only too easily from the experience of the last few years. Is it possible to maintain that when questions affecting the welfare of the great body of medical men have been before Parliament the profession has been able to pull its full weight? Was it so, for example, when the Health Insurance system was being framed? Certainly it was not. Who that cares supremely for the highest interests of the profession—and among those highest interests I rank its good repute—can look back upon the stormy events of those days without some sense of humiliation? The chaffering in public into which the profession was forced was only too well calculated to give the impression that medical men were thinking very much of their material interests and very little of the nation's welfare. Had there been in existence such a body as the Federation is fast growing to be, how different would have been the beginnings of Health Insurance! The Government would have known at the outset that it had to reckon with a Federation able to represent the views not only of doctors, but of pharmaceutical chemists (who were more immediately concerned), and also of related bodies which had no material interests at stake to deflect their judgment. How could the deliberate conclusions of such a Federation have failed to carry weight with the Government and with that public opinion which is the final court of appeal?

The need for federation is deducible not less plainly from the multiplicity of existing medical organisations. The fact that there are some two hundred separate medical bodies will surprise no one who knows how many-sided are the activities of the profession and at what a multitude of points it comes into contact with the nation's life. It certainly shows that the profession is not lacking in the organising faculty. So far, well. But is it not self-evident that the effect of all this organising energy would be vastly strengthened if these two hundred bodies were leagued together? They are influential in very different degrees. But were they all federated, the most powerful would have their influence increased tenfold; the least powerful would have their influence increased a hundredfold. These two hundred organisations may all, for aught I know, be in the highest state of efficiency, but never will they be able to speak with unimpeachable authority until they have entered into a combination which will enable them to elicit their greatest common measure of agreement. This is no case merely of counting heads. We cannot, indeed, as practical men and women, afford to disregard the weight of numbers. It is a consideration to which our law-makers are extremely susceptible, and for what it is worth we must avail ourselves of it. But I attach far more importance to the moral authority which is implicit in the deliberate conclusions reached by men and women of different yet kindred professions who have taken counsel together and can speak not only for those whose interests are directly involved in a given issue, but also for those who can claim the quality of impartiality. No conclusions so arrived at could possibly be waived by the imputation of interested motives.

### No Party Politics.

It cannot too often be insisted upon that the Federation knows no party politics. To say that it knows no politics would be absurd, for the most important of its objects is to take political action at every possible opportunity. In these days, when the scope of State action is being so rapidly enlarged, it would be strange indeed if we had no concern with politics. What is our Public Health system but a political creation? It is not the work of one political party, but of all, and it has been so manifestly free from party complexion that no political party, so far as I am aware, has ever tried to annex it as an asset. And similarly this Federation will always be detached from party politics. It is a piece of good fortune that during the first year of its existence the Federation should have had an opportunity of demonstrating *urbi et orbi* its superiority to party politics. It gave its support—its active and influential support, I will say—to Lieut.-Colonel Fremantle, who was elected as a Coalition Unionist, and to another member of the profession, Dr. Dunstan, who stood as a Labour candidate and, though

**Federation of Medical and Allied Societies**—(*continued*).

not at the top of the poll, won the second place in the list. And, as you know, every medical member of the Legislature, except Dr. Addison, whose office debars him from formal association with a movement with which he has none the less avowed his sympathy, but not excepting Lord Dawson, who can speak for us in the House of Lords, belongs to the Federation. The medical members of the House of Commons now number ten, and we know that they are only the advance guard. Before long, with the active backing of the Federation, their numbers will be multiplied, and the time is at hand when on all questions affecting health interests the Whips will have to reckon with a solid phalanx of medical men who will be able to speak with one voice on such questions, however much they may differ on other questions. No party that means honestly by the nation's health will regard such a prospect with dismay. It will obviously be to the advantage of the Government of the day, from whatever party or parties it is formed, to have the firm support of such a medical group whenever public health legislation is attempted. During the last three or four years this kind of legislation has had a free course. Money has been found for combating venereal disease, for the more effectual administration of the Health Insurance Acts, and so forth. But it may not always be so. The nation is beginning to bend and groan under its load of taxation, and the advocates of a short-sighted economy will before long have to be reckoned with. In so far as they protest against extravagance in high places we may wish them well. But if, when the economy cry has become popular, they try to starve the Public Health Service or to hinder its natural development, we must cry "Hands off!" Against any such tactics a powerful medical group in the House of Commons will be a bulwark. And behind it will stand the Federation. No longer will it be a "grave defect in medical organisation," to use Dr. Addison's words, that there should be "no body of men in the profession who could be appealed to for judgment on a great public issue." Such a body the Federation is destined to become. Nay, it will be more than that, for it will be able to declare the best mind not only of the medical but of the allied professions.

Let us not forget that we have reached an epoch in which the fountains of the great deep have been broken up. Who can fail to see that the indirect effects of the war will be as considerable, though not, we hope, so catastrophic as its direct effects? We have seen the direct effects in overturned thrones, in dissolved empires, in rolled-up maps. The indirect effects are coming upon us with no laggard steps, and among them is an urgent call to the medical profession, as to every other section of the community, to adapt itself to the clamorous needs of a new age. The go-as-you-please style, with which the profession has hitherto been content, is now frankly impossible. Medical education must be reformed, the hospitals problem must without loss of time be grappled with, a linking-up and reorganisation of medical arrangements generally can no longer be postponed. That the system known as State medicine will undergo much further development is inevitable. Some hold, indeed, that the whole profession must be pressed into the service of the State—that medicine must, in other words, be nationalised. Others regard such a consummation with horror, as destructive of initiative and tending to reduce practitioners to the status of mere cogs in a huge machine. I have my views on that question, but it is not for me, as your Chairman, to declare them. What I do say is that the profession has no alternative but to adapt itself to the nation's needs, whatever form the adaptation may take. That task will require more statesmanship than is to be found in any one of the existing medical organisations, be it never so powerful. It is an enterprise which will tax to the uttermost the statesmanship of the whole profession. If that be so, how can any medical society or association be justified in withholding whatever contributions it can make to the common stock? Is it not clear that nothing less than federation will enable the greatest of all the professions adequately to fulfil its duty to the nation?

The Position of Constituent Societies.

Finally, I protest, with all the emphasis at my command, that the Federation is absolutely innocent of any design to supersede the independent action of its constituent bodies. Every such body preserves its full powers of self-determination, and is as free to take individual action on any question as it was before joining the Federation. Naturally, having entered the Federation, it will present for discussion the views which it entertains on any matter of common interest. But whether those views secure the concurrence of the Federation or not, it will be absolutely free to take whatever separate action it pleases. The Federation's first and greatest function is that of co-ordination. Its conferences will all be round-table conferences, in which no priority or predominance can or will be claimed by any. So far from running any risk of absorption, each of the constituent bodies will have its independence established on a more unassailable basis than ever; it will be able to justify its existence in the eyes of those to whom it is responsible by pointing to the results achieved by the allied action in which it has participated, results which will be far superior to any that could have been accomplished by its isolated efforts. The Federation, I boldly assert, has no prejudices, no rivalries, no antagonisms.

